# Stem cell laden nano and micro collagen/PLGA bimodal fibrous patches for myocardial regeneration

**DOI:** 10.1186/s40824-022-00319-w

**Published:** 2022-12-13

**Authors:** Jung Hee Wee, Ki-Dong Yoo, Sung Bo Sim, Hyun Joo Kim, Han Joon Kim, Kyu Nam Park, Gee-Hee Kim, Mi Hyoung Moon, Su Jung You, Mi Yeon Ha, Dae Hyeok Yang, Heung Jae Chun, Jae Hoon Ko, Chun Ho Kim

**Affiliations:** 1 Department of Emergency Medicine, Yeouido St. Mary’s Hospital, College of Medicine, The Catholic University of Korea, Seoul, 07345 Republic of Korea; 2 Division of Cardiology, Department of Internal Medicine, St. Vincent’s Hospital, Suwon, 16247 Republic of Korea; 3 Department of Thoracic and Cardiovascular Surgery, Bucheon St. Mary’s Hospital, College of Medicine, The Catholic University of Korea, Bucheon, 14647 Seoul, Republic of Korea; 4 Institute of Cell and Tissue Engineering, College of Medicine, The Catholic University of Korea, Seoul, 06591 Republic of Korea; 5 Department of Emergency Medicine, The Catholic University of Korea, Seoul, 06591 Republic of Korea; 6 Department of Thoracic and Cardiovascular Surgery, Seoul St. Mary’s Hospital, College of Medicine, The Catholic University of Korea, Seoul, 06591 Republic of Korea; 7 Department of Medical Life Sciences, The Catholic University of Korea, Seoul, 06591 Republic of Korea; 8 Department of Biomedicine & Health Sciences, College of Medicine, The Catholic University of Korea, Seoul, 06591 Republic of Korea; 9 Smart Textiles R&D group, Korea Institute of Industrial Technology (KITEC), Ansan, 31056 Republic of Korea; 10 Laboratory of Tissue Engineering, Korea Institute of Radiological and Medical Sciences, 01812 Seoul, Republic of Korea

**Keywords:** Nano and micro bimodal fibrous scaffold, Cardiac patch, Collagen, PLGA, BMSCs, Myocardial regeneration

## Abstract

**Background:**

Although the use of cardiac patches is still controversial, cardiac patch has the significance in the field of the tissue engineered cardiac regeneration because it overcomes several shortcomings of intra-myocardial injection by providing a template for cells to form a cohesive sheet. So far, fibrous scaffolds fabricated using electrospinning technique have been increasingly explored for preparation of cardiac patches. One of the problems with the use of electrospinning is that nanofibrous structures hardly allow the infiltration of cells for development of 3D tissue construct. In this respect, we have prepared novel bi-modal electrospun scaffolds as a feasible strategy to address the challenges in cardiac tissue engineering .

**Methods:**

Nano/micro bimodal composite fibrous patch composed of collagen and poly (D, L-lactic-co-glycolic acid) (Col/PLGA) was fabricated using an independent nozzle control multi-electrospinning apparatus, and its feasibility as the stem cell laden cardiac patch was systemically investigated.

**Results:**

Nano/micro bimodal distributions of Col/PLGA patches without beaded fibers were obtained in the range of the 4-6% collagen concentration. The poor mechanical properties of collagen and the hydrophobic property of PLGA were improved by co-electrospinning. *In vitro* experiments using bone marrow-derived mesenchymal stem cells (BMSCs) revealed that Col/PLGA showed improved cyto-compatibility and proliferation capacity compared to PLGA, and their extent increased with increase in collagen content. The results of tracing nanoparticle-labeled as well as GFP transfected BMSCs strongly support that Col/PLGA possesses the long-term stem cells retention capability, thereby allowing stem cells to directly function as myocardial and vascular endothelial cells or to secrete the recovery factors, which in turn leads to improved heart function proved by histological and echocardiographic findings.

**Conclusion:**

Col/PLGA bimodal cardiac patch could significantly attenuate cardiac remodeling and fully recover the cardiac function, as a consequence of their potent long term stem cell engraftment capability.

**Supplementary Information:**

The online version contains supplementary material available at 10.1186/s40824-022-00319-w.

## Introduction

Ischemic heart disease is commonly caused by coronary atherosclerosis that hardens and narrows arteries. These arteries slow down the blood flow to myocardium, leading to myocardial infarction, cardiac dilation, or even death. Despite the advances in drugs and medical technologies, ischemic heart disease is recognized as the primary cause of death globally, because the adult cardiomyocyte lacks self-renewal capacity [1, 2]. Over the last decades, stem cells have been spotlighted as alternative source of myocardium because of their pluri- or multipotency; therefore, a myriad of experimental and clinical efforts based on stem cells have made to achieve structural and functional repair of injured myocardium [3, 4]. Many of these studies have demonstrated the promising results, however, the extent of benefits of these cellular therapeutics remain insufficient. A common reason for this is the low engraftment rate of transplanted stem cells. Previously performed stem cell delivery for cardiovascular research mostly dependent upon the simple injection delivery system have demonstrated only 5~10% of the initial retention rate because the normal highly organized architecture of extracellular matrix for cell engraftment disappears in the infarct region [5, 6]. In addition, even barely engrafted cells showed poor survival rates because of the lack of oxygen and nutrient supply [7]. To address these issues, cardiac tissue engineering using scaffold has emerged as a new strategy. Three-dimensional scaffolds made of biomaterials may provide an architectural context in which stem cells adhere, migrate, proliferate, and differentiate, similar to natural extracellular matrix [8–10].

Collagen, as the main structural protein in the extracellular space in the various connective tissues, can be a suitable material for manufacturing the scaffold for cardiac tissue engineering. Collagen constitutes around 33% of the total myocardial protein and has abundant motifs in its helix that play a crucial role in cell adhesion, migration and proliferation, essential for stem cell retention [11, 12]. Beside the biocompatibility, the stiffness and the elasticity of scaffold can be important factors determining stem cell fate. Because of repeated myocardial contraction and relaxation, the biomaterials for myocardial regeneration must have elasticity and mechanical strength to withstand the dilation of the ventricles [13]. However, collagen, similar to other naturally derived biomaterials, has brittle mechanical characteristics. Therefore, to overcome the drawback of poor mechanical properties, the preparation of composite materials that utilize flexible synthetic polymers along with collagen can be one of the recommendations for the development of new scaffold for cardiac tissue engineering [14]. As FDA approved aliphatic biodegradable polyesters, poly(lactic acid) (PLA), poly(glycolic acid), and their copolymer, poly(lactic-co-glycolic acid), (PLGA) can be the suitable candidate materials for manufacturing the composite scaffold, among which, PLGA is particularly attractive, because bulk properties and biodegradation rate can be controlled by its copolymerization [15].

So far, nanofibrous scaffolds prepared by electrospinning have been recognized to have potential in this field because of their structural similarity to natural ECM. However, they have demonstrated reduced porosity and have inhibited cellular penetration into the construct, preventing 3D tissue formation. Here, we propose a novel hybrid scale scaffold consists of nano and microfibers that provide the significantly increased pore size and reinforced mechanical strength compared with nanofibers alone. Microscale PLGA fibers have the advantage of forming 3D ECM-mimicking structures, whereas nanoscale collagen fibers mimic the structure of the ECM fibers for focal adhesion that orchestrates various aspects of cellular behaviors for the development and maintenance of tissues. To take these advantages of hybrid scale scaffold, we fabricated collagen and PLGA nano and micro bimodal fibrous scaffolds for cardiac regeneration and repair through an independent nozzle control with patented multi-electrospinning instrument [16]. This fibrous scaffold integrated with bone marrow-derived mesenchymal stem cells (BMSCs) was applied to rabbit MI model, and the feasibility as a tissue engineered cardiac patch was systemically investigated.

## Material and method

### Electrospinning

Fabrication of the electrospun collagen/PLGA nano and micro fibrous patch was carried out using the independent multi-nozzle electrospinning technique first employed by H. K. Park et al. [16]. PLGA and collagen were spun using 10 wt% PLGA (Neosorb,10/90, Mw: 200 kDa, Samyang Biopharmaceuticals Co. Ltd., Seongnam, Korea) dissolved in HFIP (1,1,1,3,3,3-Hexafluoro-2-propanol, Acros Organics, Pittsburgh, PA, USA), and 9 wt% succinated atelocollagen (Type I, Mw: 220 kDa, Dalim Tissen Co., Seoul, Korea) solution dissolved in HFIP at 25 kV power and 4 mL/h 21G needle and 15 kV power and 2 mL/h flow rate, respectively, as described previously. In the case of Col/PLGA, the concentration of collagen solution was lowered to 4, 5, and 6 wt% and spun at 25kV power and 1.2 mL/h flow rate to avoid the formation of collagen beaded fibers. The thickness of electrospun fiber samples were 500±10μm.

The Col/PLGA samples were immersed into a 1-ethyl-3-(3-dimethylminopropyl) carbodiimide (Sigma-Aldrich, MO, USA) and N-hydroxysuccinimide (Sigma-Aldrich, MO, USA) solution of 0.6 M for 12 h consecutively for crosslinking. After crosslinking, the samples were dried at room temperature and washed three times with MilliQ water to remove the residual chemicals.

### Morphology analysis

The surface morphology of the samples was observed using an SEM (JSM-5510, JEOL Ltd., Tokyo, Japan). The samples were gold-coated using a sputter-coater (Bio-Rad Microscience Ltd., Hert-fordshire, UK) with an accelerating voltage of 2 kV prior to SEM observation. The average diameters of the fibers in the samples were calculated by an image analysis program (Scope Eye II) with the randomly selected 100 fiber strands.

### Surface wettability

The surface wettability of Col/PLGA was examined by static contact angle (G-1, Erma Inc., Tokyo, Japan) measurements and compared to those of the PLGA and Col samples. The measurement was carried out with an initial drop volume of 25 mL as a function of time.

### Mechanical properties

The uniaxial tensile properties of the samples were measured at room temperature with a universal testing machine (UTM 4476, Instron, Norwood, MA, USA). In accordance with ASTM D-638-5, the samples were stretched to breaking point under a constant crosshead speed of 10 mm/min and chart speed of 20 mm/min, using 5-kgf load cells.

### Isolation of rabbit BMSCs

Isolation and cultivation of the rabbit BMSCs were accomplished by institute of Hansen’s disease, Catholic university. Animal experimental protocols were approved by the Institutional Animal Care and Use Committee (IACUC) in College of Medicine, the Catholic University of Korea (CUMC-2010-0034-01). Briefly, bone marrow cells were aspirated from the femur and tibia of 4 weeks old New Zealand White rabbits (Orient Bio Inc., Seongnam, Korea) and re-suspended in phosphate-buffered saline (PBS; Biowest, Nuaillé, France). The Collected cells were slowly layered above FiColl-Paque PLUS (GE Healthcare, Amersham Biosciences, UK), for gradient centrifugation at 400×g for 30 min. After centrifugation, bone marrow cells were separated over the gradient interface. These cells are aspirated and rinsed with PBS. Subsequently, these bone marrow cells were cultured with low glucose Dulbecco's Modified Eagles Medium (DMEM; Biowest, Nuaillé, France) containing 10% fetal bovine serum (FBS; Biowest, Nuaillé, France) and 1% penicillin streptomycin solution (Biowest, Nuaillé, France) at 37 °C in humidified atmospheres with 5% CO_2_. After overnight incubation, the non-adherent cells were removed by replacing the medium. Thereafter, the culture medium was replaced every 3 days. At 80-90% confluence, cells were sub-cultured to passage 7 by using culture medium.

### Identification of BMSCs

BMSCs were trypsinized at 80% confluence for phenotypic characterization using flow cytometry (FACSCanto II, BD Biosciences, San Jose, CA, USA). The cells were resuspended in PBS at a concentration of 20,000cells/mL. Resuspended cells were transferred into round-bottom polystyrene tube (Thermo Fisher Scientific, Waltham, MA, USA), and then incubated with antibodies of CD 14 (AbD Serotec, Kidlington, UK), CD 29 (Abcam, Cambridge, UK), CD 44 (Abcam, Cambridge, UK) and CD 45 (AbD Serotec, Kidlington, UK) in PBS/0.1% bovine serum albumin (BSA) for 30 min at room temperature. Then, the cells were stained with secondary antibodies for 1 h. The secondary antibodies were used following with Alexa Fluor® Plus 488 (Invitrogen, Thermo Fisher Scientific, Inc., MA, USA). Prior to flow cytometric analysis, cells were washed using PBS. All data were acquired with an event counting set for 10,000 cells. The data were analyzed with Cell Quest software (BD Biosciences, San Jose, CA, USA).

### GFP-tracking BMSCs

In order to trace the distribution of transplanted BMSCs, cells were labeled with GFP. BMSCs were seeded into cultured plates at the density of 2 × 10^6^ cells/mL. The following day, the BMSCs were transfected with GFP expression lentivirus (GFP (CMV-Bsd) lentiviral particles, Gentarget Inc. CA. USA) according to the manufacturer’s protoCol. For optimal transduction efficiency, 8 μg/mL of polybrene were added. After 72 hours, transduced BMSCs were washed and changed with fresh medium. GFP-labeled BMSCs (GFP-BMSCs) were evaluated using florescent microscope (Axiovert 200, Zeiss, Germany), and transduction efficiency was determined by flow cytometry.

### Cell seeding, proliferation and cell cytotoxicity on the patch

The patches were cut with a diameter of 1.2 cm, and then sterilized with ethylene oxide gas. BMSCs were seeded at a concentration of 1 × 10^6^ cells/patch for in vivo experiment and 1 × 10^4^ cells per patch for in vitro proliferation experiment, and each of the patches was filled with 500μL of fresh media. The BMSCs-seeded patches were cultured under static conditions for 2 days before *in vivo* experiment, and for 7 days for *in vitro* experiment. The culture medium was exchanged every 2-3 days.

Proliferation of BMSCs on patches was assessed using the cell counting kit (CCK-8; Dojindo Laboratories, Kumamoto, Japan). At each time point, cell-seeded patches were transferred to a fresh medium containing 10% (v/v) CCK-8 reagent. After 4 hours incubation, 200 μL of media was obtained for measurement of absorbance. The absorbance was determined at 450 nm in micro-plate reader (PowerWave XS, Bio-TEK)

Cell cytotoxicity of BMSCs on patches was determined using LIVE/DEAD assay kit (Invitrogen, Thermo Fisher Scientific, Inc., MA, USA). On day 7, the medium was removed from each well and the BMSCs on patches were washed with PBS. The BMSCs were then incubated for 1 h with a 500 μL solution containing 2 μM calcein AM and 4 μM ethidium homodimer-1 dissolved in PBS. The viable cells (green fluorescence) and necrotic cells (red fluorescence) were visualized using a fluorescence microscope.

### Rabbit myocardial infarction model and surgical implantation of BMSCs laden cardiac patch

**Scheme 1:**
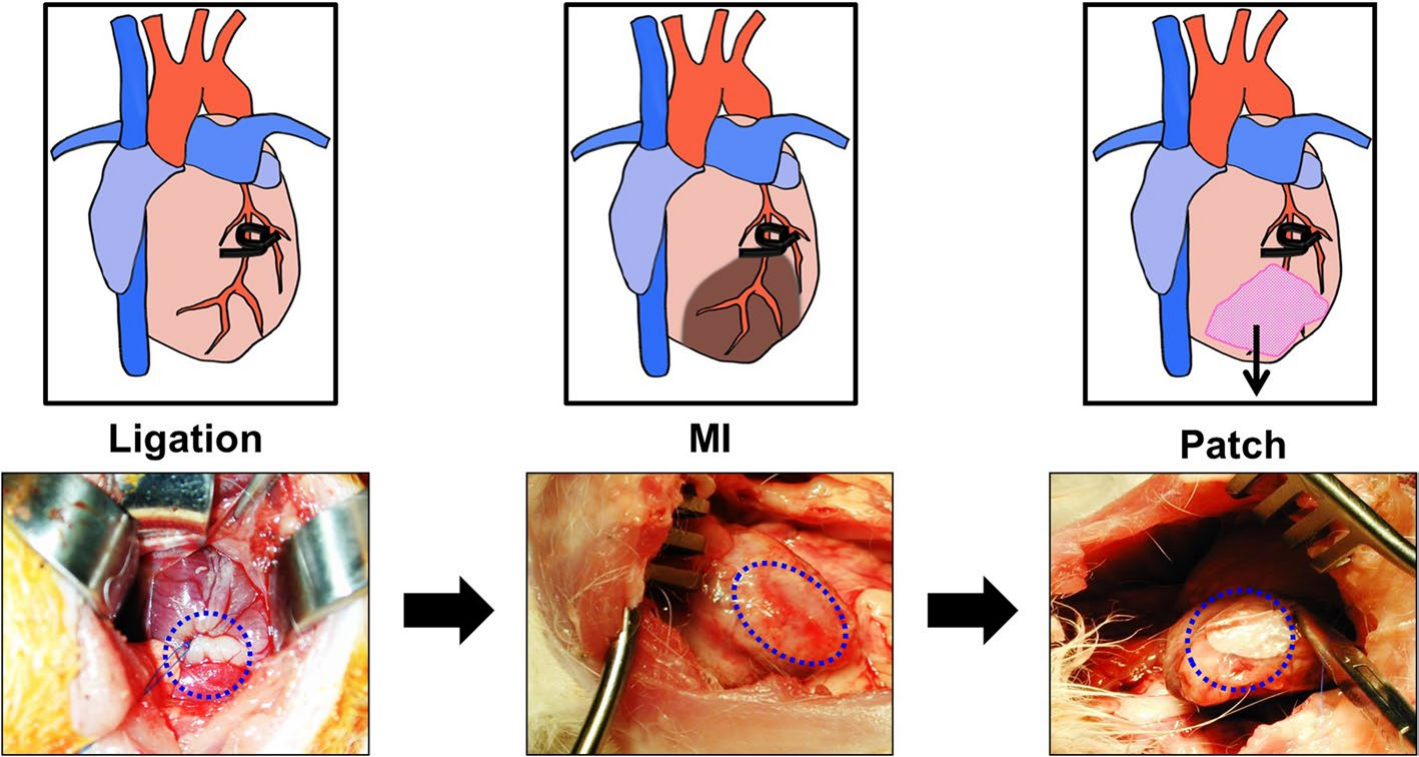
Schematic illustrations and representative images of the patch implantation after MI in rabbits

Animal experimental protocols were approved by the Institutional Animal Care and Use Committee (IACUC) in College of Medicine, the Catholic University of Korea (CUMC-2012-0086-01). Male New Zealand White rabbits weighing 3.0-3.5kg were used in this study, and randomly assigned to rabbit without MI induction (sham-operated group), rabbit with MI induction (MI group), and rabbit with MI induction treated with BMSCs laden cardiac patch transplantation (patch group), n=5 respectively. Rabbit was fasted for 6 hours before surgery and anesthetized with Zoletil® (Virbac, Carros, France) and Rompun® (Bayer, Leverkusen, Germany) by intramuscular injection. For deep anesthesia, additional inhalation was performed by tracheal intubation. The rabbit heart was exposed via a left 4th intercostal thoracotomy. The pericardium was incised and the left anterior descending artery (LAD) was identified and proximally ligated using 4-0 prolene suture (Ethicon, NJ, USA). After the intercostal suture, the muscle and skin were sutured sequentially after reestablishing the negative pressure in the chest cavity. The permanent MI induction was assessed through the echocardiography. The transplantation procedure was performed 4weeks post-MI. The heart of MI rabbit was exposed by left 4th intercostal thoracotomy according to the previous incision, and cardiac patches with BMSCs were sutured along the margin of the MI area to cover the defect created in the left ventricle (Scheme 1). Each of the four sides of the patch was sutured in a simple interrupted suture pattern and attached to the infarct area. After implantation, the negative pressure was restored and then sutured.

### *In vivo* imaging of nanoparticles-labeled BMSCs

The transplanted BMSCs observed cell migration using fluorescent nanoparticles. BMSCs were labeled with NEO-STEM™ nanoparticles (Biterials, Seoul, South Korea) according to the manufacturer's protocol. Briefly, Cells were seeded in T-75 flasks at densities of 1 × 10^6^ cells/flask, and incubated with the suspension of nanoparticles (0.2 mg/mL) for 24 hours. After incubation, labeled BMSCs were washed by PBS and then, cultured at 1 × 10^4^ cells on the patch for 3 days. Patches containing labeled BMSCs were implanted into rabbit MI. To examine the distribution of BMSCs, hearts were extracted at 4 weeks, and then images were obtained using Maestro™ EX in vivo imaging system (CRi. Inc., MA, USA).

### Histology and immunohistochemistry

After 4 weeks of BMSCs transplantation, the rabbits were sacrificed with carbon dioxide atmosphere under anesthesia and their hearts were removed and sliced by transverse section. Before the sectioning process, remain patches has been carefully removed before embedding in order to obtain 4-5 μm thickness of fine tissue section. After washing with saline, the hearts were fixed in 10% formalin for overnight and then embedded in paraffin. Hematoxylin and eosin and Masson trichrome staining were performed for histopathological evaluation. To measure the infarct size, the heart was cut into four sections transversely, and the infarct length and thickness of the sections were analyzed by Image J [17].

Immunohistochemistry was also employed to assess the cardiac regeneration with following antibodies; green fluorescent protein, GFP (Abcam, Cambridge, UK), cardiac troponin-I, cTnI (Abcam, Cambridge, UK), α-smooth muscle, Acta2 (Abcam, Cambridge, UK), N-cadherin (Sigma-Aldrich, MO, USA), connexin-43 (Sigma-Aldrich, MO, USA). The sections were deparaffinized and hydrated, and then boiled with 0.1 mol/L citric acid buffer. Blocking buffer (0.25% Triton X-100, 5% bovine serum albumin, PBS) was treated on the samples for 60 minutes at room temperature. The sections were incubated with primary antibodies for overnight at 4 °C. After incubation, the sections were washed, followed by incubation with secondary antibodies (DyLight^TM^ 594 conjugated highly cross-adsorbed and DyLight^TM^ 488 conjugated highly cross-adsorbed; Thermo Fisher Scientific, Waltham, MA, USA) for 2 hours at room temperature. 4′,6′-diamindino-2-phenylindole (DAPI, Thermo Fisher Scientific, Germany) was used to visualize nuclei. Images were obtained with confocal microscope (LSM800 with Airyscan, Zeiss, Germany).

### Terminal deoxynucleotidyl transferase dUTP nick-end labeling (TUNEL) Assay

Apoptotic cardiomyocytes in heart tissue were detected with a terminal deoxynucleotide transferase-mediated dUTP nick-end labeling (TUNEL) assay (*In Situ* Cell Death DetectionKit AP, Roche Applied Science, Mannheim, Germany) according to the manufacturer instructions. In brief, the deparaffinized myocardial tissue sections were heated in sodium citrate solution and digested with proteinase K for 15 min at room temperature. The sections were incubated with terminal deoxynucleotidyl transferase for 1 h at 37 °C. After TUNEL staining, the specimens were counterstained by with 4, 6-diamidino-2-phenylindole (DAPI). TUNEL stained cells was observed under a fluorescence microscope.

### Quantitative real-time PCR

Total RNA was extracted from cardiac tissue using TRIzol Reagent (Invitrogen, Thermo Fisher Scientific, Inc., MA, USA). TRIzol treated samples were homogenized, and then chloroform was added. After centrifugation, upper aqueous phase was transferred into a new tube. The aqueous phase containing the RNA was precipitated by isopropanol. The RNA pellet was air-dried and dissolved in nuclease-free water. RNA quantity was assessed by NanoDrop^TM^2000 (Thermo Fisher Scientific, Waltham, MA, USA). Reverse transcription was carried out with SuperScript™ IV First-Strand Synthesis System (Invitrogen, Thermo Fisher Scientific, Inc., MA, USA), following the manufacturer’s instruction. To assess the gene expression levels, real-time PCR was performed using iQ SYBR Green Supermix (Bio-Rad, Hercules, CA, USA) on a CFX96TM Real-Time system (Bio-Rad, Hercules, CA, USA). Glyceraldehyde-3-phos-phate dehydrogenase (GAPDH), forward 5′-ggaatccactggcgtcttca-3′ and reverse 5′-tacttctcgtggttcacgcc-3′, was used as an internal control, and PCR primers used are as follows; VEGF, forward 5′-ggagtaccctgatgagatcga-3′ and reverse 5′-ctttggtctgcattcacatttgt-3′; TGF-beta, forward 5′-cggcagctgtacattgactt-3′ and reverse 5′-arcgcacgatcatgttggac-3′; vWF, forward 5′-gtgaactcgcagtgtgcggac-3′ and reverse 5′-aggtctgcagcagttcatcc-3′; myocardin, forward 5′-ttgggaaacaatggagtg-3′ and reverse 5′-ttgagatccgtgacatcc-3′; SM22 alpha, forward 5′-aacaaatggagcaggtgg-3′ and reverse 5′-tgccaggctgcccaaa-3′; GATA-4, forward 5′-agagagtgtgtcaactgcgg-3′ and reverse 5′-gatgaggggccggttgatg-3′. Data analysis was performed by the 2-^ΔΔ^Ct method.

### Echocardiography

Echocardiography was performed with a Squoia C256 system (Siemens, California, USA), which is equipped with a phased-array 15 MHz. Intra-muscular injections of ketamine (35 mg/kg) and midazolam (0.9 mg/kg) were used for sedation. The echocardiography examinations were performed at baseline and weeks 4 to assess the LV morphology and function of the left ventricle by measuring the volume of the systole and the diastole and calculating the left ventricular fractional shortening (LVFS, %) and left ventricular Ejection fraction (LVEF, %). In the left parasternal long axis view and the left parasternal short axis view of rabbits, the two-dimensional echocardiography and the M mode echocardiogram was performed in a section near vertical at the left ventricular papillary muscle. All the examination contents were recorded as digital images and recorded as Digital Imaging and Communications in Medicine (DICOM) offline. After measuring the left ventricular end-systolic internal dimension (LVIDs) and the left ventricular end-diastolic internal dimension (LVIDd) by the method using M-mode suggested by the American Society of echocardiography, LVFS and LVEF were obtained as follows:

$$\mathrm{LVFS}(\%)=\left\{(\mathrm{LVIDd}-\mathrm{LVIDs})/\mathrm{LVIDd}\right\}\;\times\;100\;,\;\mathrm{LVEF}\;(\%)=\left\{{(\mathrm{LVIDd})}^2-{(\mathrm{LVIDs})}^2/{(\mathrm{LVIDd})}^2\right\}\times100.$$

### Statistical analysis

All experiments data were expressed as the mean ± standard deviation (SD). Statistical analysis was performed using the SPSS 19.0 software package (SPSS, IBM, USA). Significance differences was measured using one-way analysis of variance (ANOVA) and Student’s t-test (*P < 0.05, **P < 0.01). *P* < 0.05 was considered statistically significant.

## Results

### Surface characteristics

**Fig. 1:**
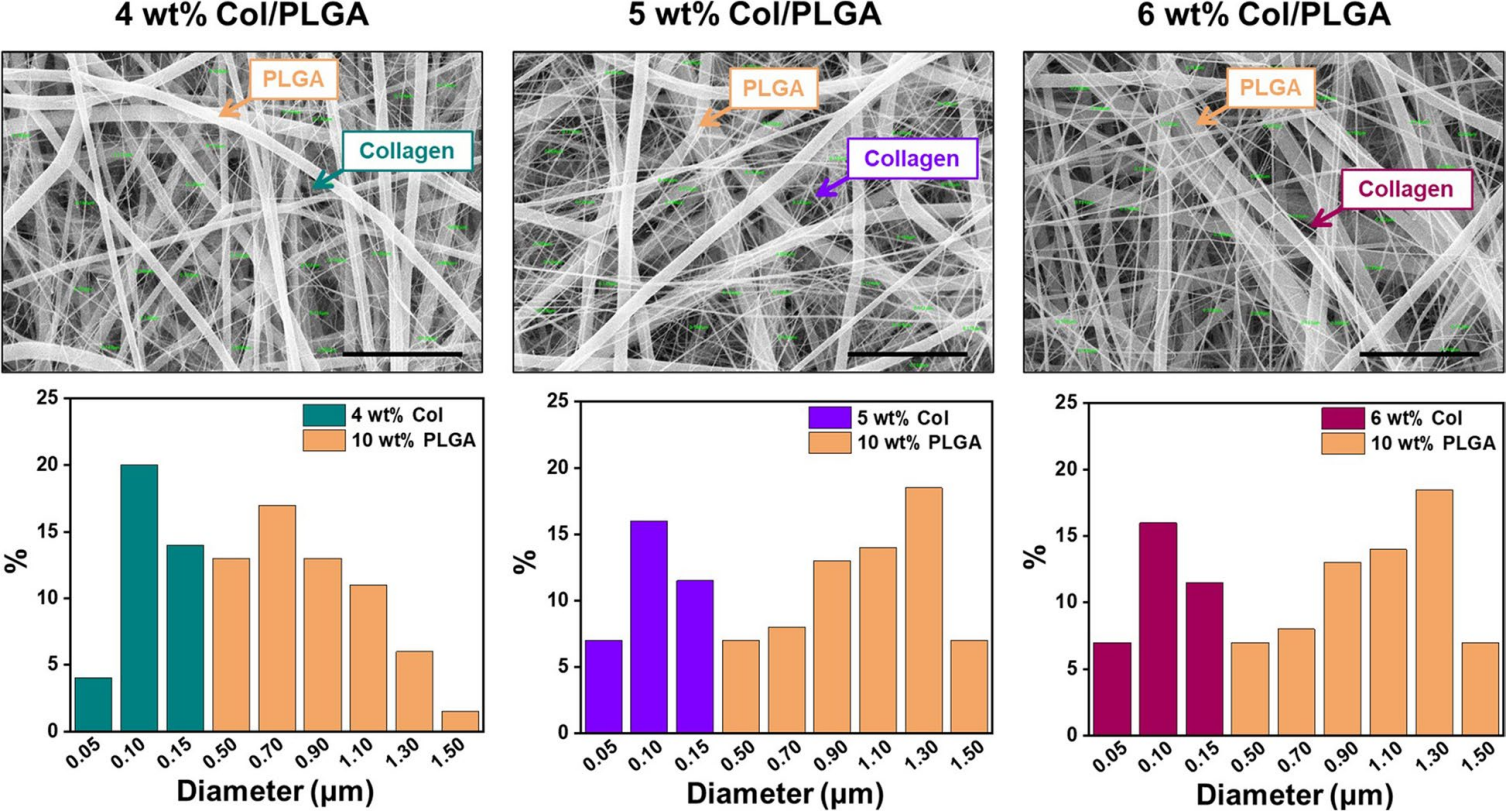
SEM images and size distribution of Col/PLGA samples (Scale bar, 10 μm)

Figure 1 shows the SEM images of Col/PLGA patches and their fiber diameter distributions, respectively. The diameter of the PLGA fibers ranges from 0.5 and 1.5 μm in all samples, whereas, the diameter of collagen fibers is in the range of 50-250 nm. Consequently, a series of Col/PLGA fibrous scaffolds composed of nanoscale smooth and continuous collagen fibers and microscale PLGA fibers with the bimodal distribution were obtained.

Figure S1 shows the SEM images of the electrospun collagen fiber structures with respect to the concentration of collagen. 3wt % collagen (Fig. S1a) could be the representative image of beaded collagen fibers spun with low concentration of collagen solution below a range of viscosity; the entanglement concentration the electrospinning process led to non-continuous and collected fibers resulted in rich beads with structural imperfections [18]. These defects were not evident in collagen patches obtained by electrospinning of solutions above the entanglement concentration, and continuous nanofibers were obtained from collagen solutions greater than 4 wt%. Meanwhile, a sudden increase in fiber diameter was found when the concentration of collagen solution was more than 7 wt%, as shown in Fig. S1e. Therefore, in this study, 4-6% of collagen concentration was set as the optimal concentration range.

**Fig. 2:**
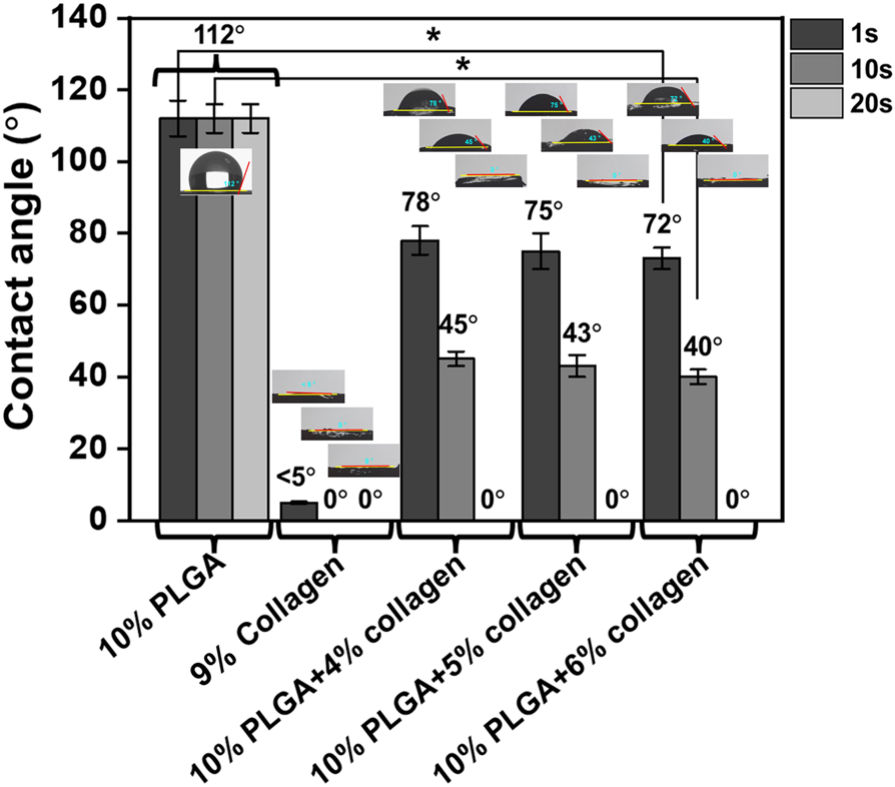
Water contact angles of the samples. The error bars represent the mean ± standard deviation (*n* = 3). This experiment was repeated three times (*p < 0.05)

Figure 2 shows the static contact angles of PLGA, collagen, and Col/PLGA patches as a function of time. The PLGA patch had a water contact angle of about 110° when 25 mL of water was first dropped, and the angle hardly changed over time. On the contrary, on the surface of the collagen patch, the water droplet had an initial contact angle of less than 5 degrees due to the strong hydrophilicity and was absorbed instantly. Meanwhile, the contact angle of the initial droplet on the surface of Col/PLGA patch decreased to 78~72° as the collagen content increased, and then the water spread and completely soaked through the fibrous structure of the samples within 20 seconds. This indicates that the hydrophilicity of Col/PLGA patch was strongly dominated by the presence of collagen.

### Mechanical property

**Fig. 3:**
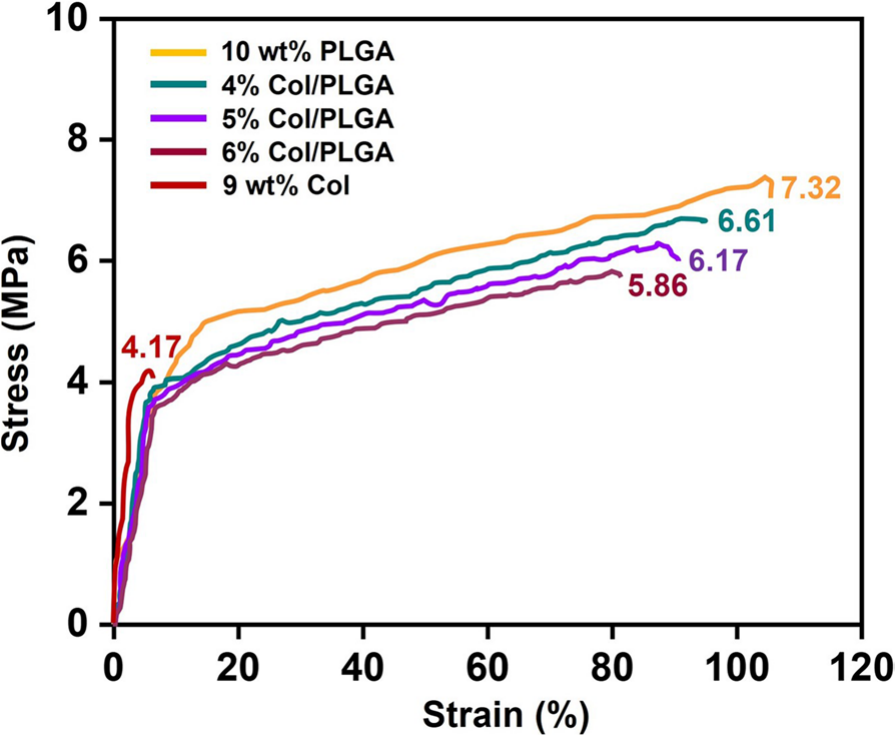
Stress-strain curves of the samples with respect to the compositional change of collagen; measured at room temperature using 5-kgf load cells. The measurement was conducted under a constant crosshead speed of 10 mm/min and chart speed of 20 mm/min

Figure 3 shows the stress-strain curve of the patches with respect to the compositional change of collagen. Both tensile strength and elongation decreased as the content of collagen increased, but the differences were not significant and all the samples showed the typical stress-strain curve of the plastics. In the end, it is strongly suggested that the preparation of composite materials that utilize flexible synthetic polymers along with collagen overcomes its poor mechanical properties [19].

### Phenotypic characterization of BMSCs

**Fig. 4:**
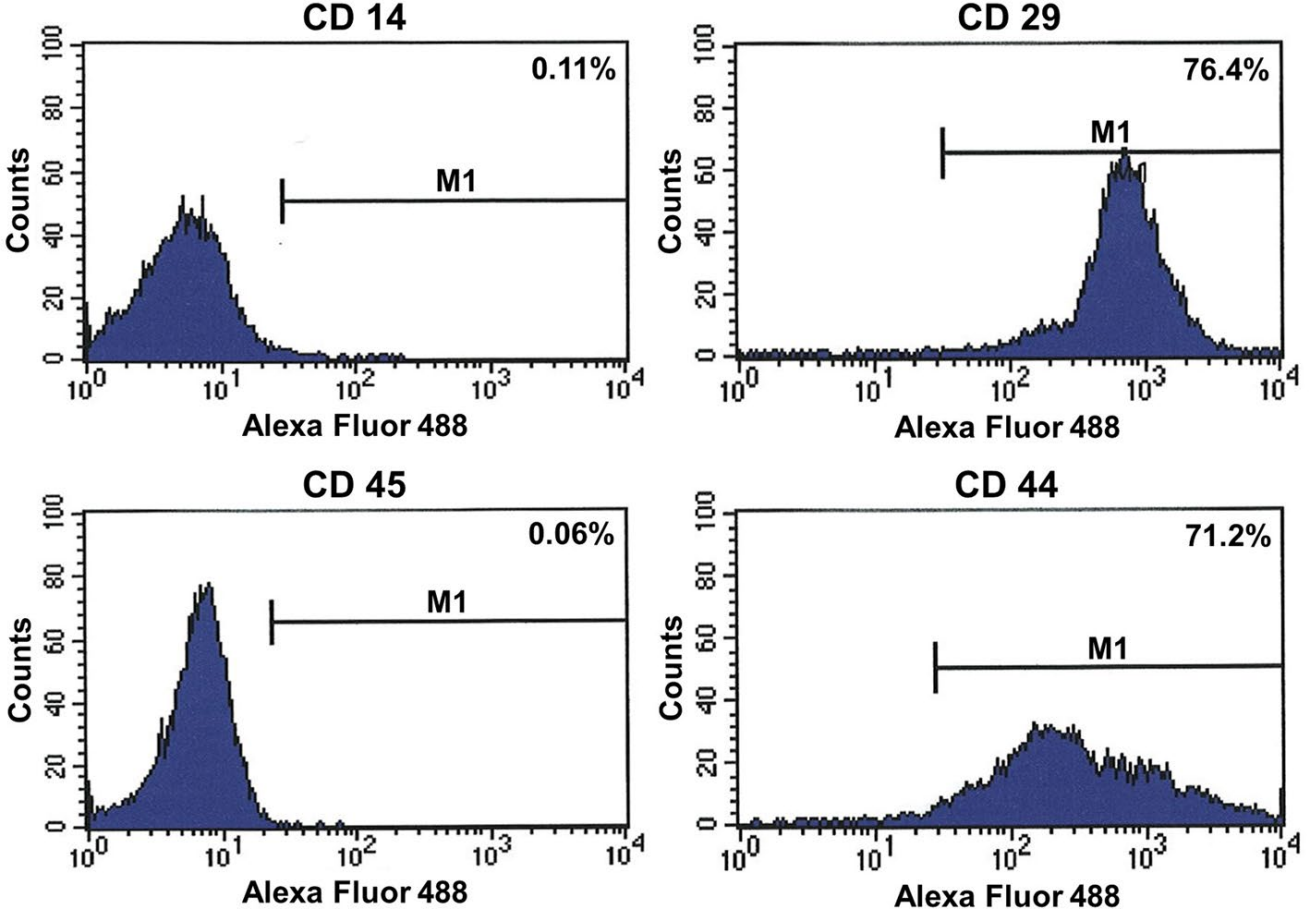
Phenotypic characterization of BMSCs using flow cytometry

The immunophenotype of the BMSCs was investigated by quantitative flow cytometry (Fig. 4). All BMSCs were negative for typical hematopoietic markers, including CD14 and CD45 while they were positive for CD29 and CD44 as previously described [20]. Therefore, this result was suggested to be sufficient for the identification of BMSCs.

### Labeling with GFP-lentiviral particles

**Fig. 5:**
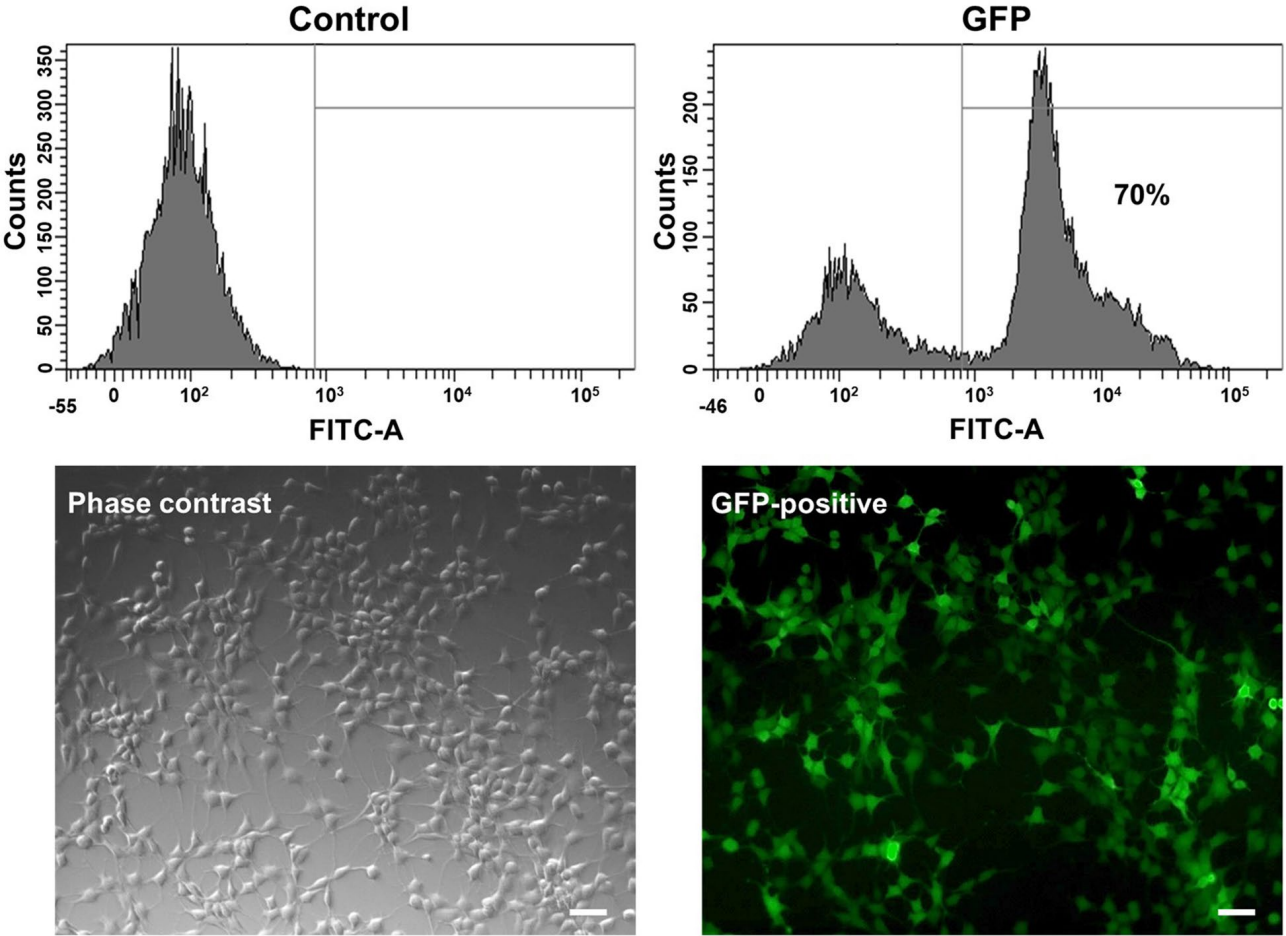
GFP-lentiviral vector transduction into BMSCs. Analysis of infection efficiency was performed by flow cytometry. Fluorescence microscope was used to detected GFP expression in the BMSCs transfected with lentiviral vector (Scale bar, 50 μm)

In order to trace the engraftment and/or differentiation of the transplanted cells, BMSCs were labeled with GFP. GFP could be monitored within target without interference from reagent introduction [21]. Quantification of GFP-positive cells by flow cytometry demonstrated a transduction efficiency of nearly 70% (Fig. 5).

### Cytotoxicity and proliferation

**Fig. 6:**
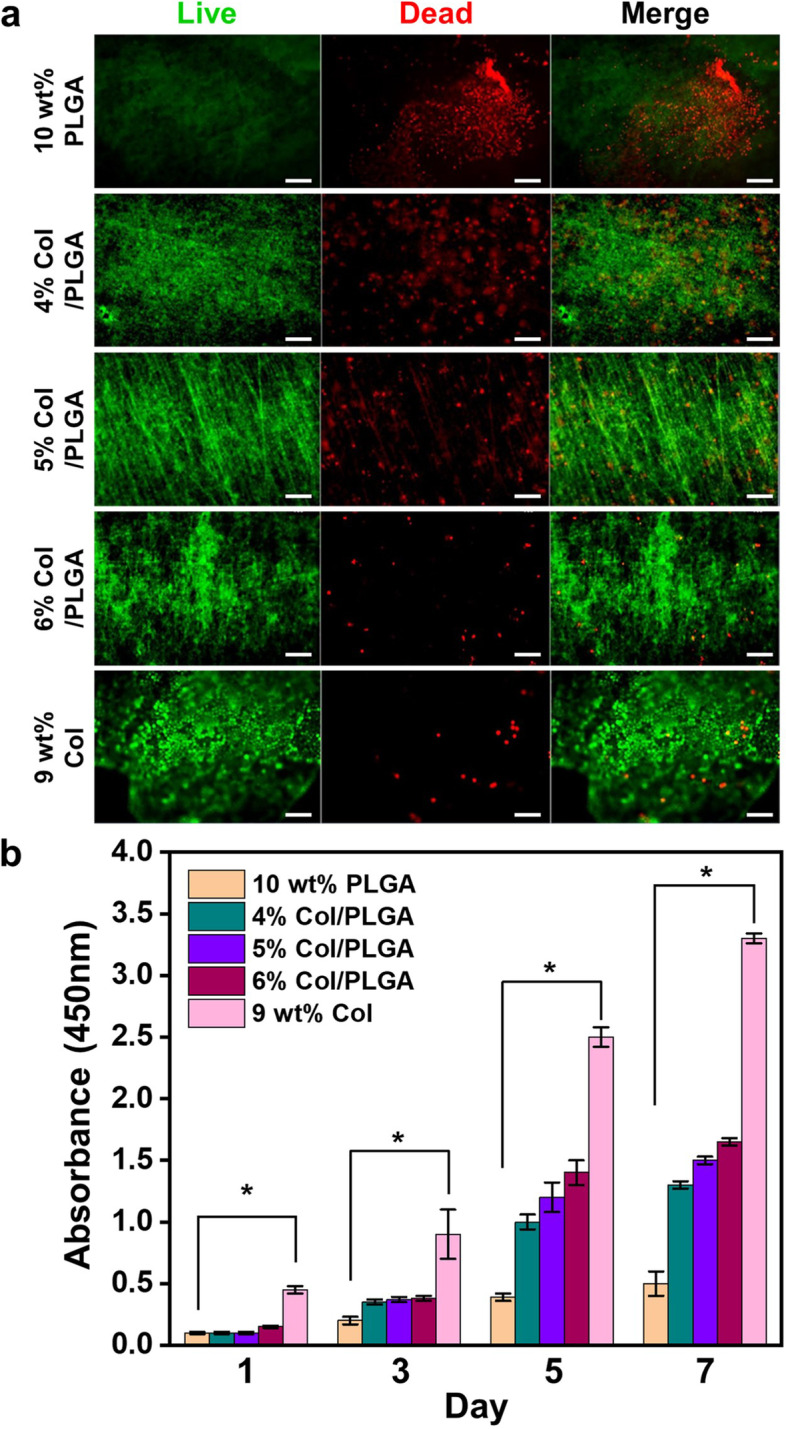
Live/dead fluorescence images (**a**) and CCK-8 assay (**b**) for BMSCs proliferation in the samples (Scale bar, 100 μm) (*p < 0.05)

The fluorescence images of the prepared fibrous patches are represented in Fig. 6a. Live cells appeared in green, and dead cells appeared in red. Following 7 days of culture, Col/PLGA patches showed higher live cell densities than PLGA patch, and live cell density increased with increased in the content of collagen. In addition, BMSCs proliferation was tested using CCK-8 assay for up to 7 days (Fig. 6b). The proliferation rates of BMSCs on Col/PLGA patches were increased gradually over time. Noticeably, 6% Col/PLGA patch exhibited the highest BMSCs proliferation among Col/PLGA patches.

### Histological assessment

**Fig. 7:**
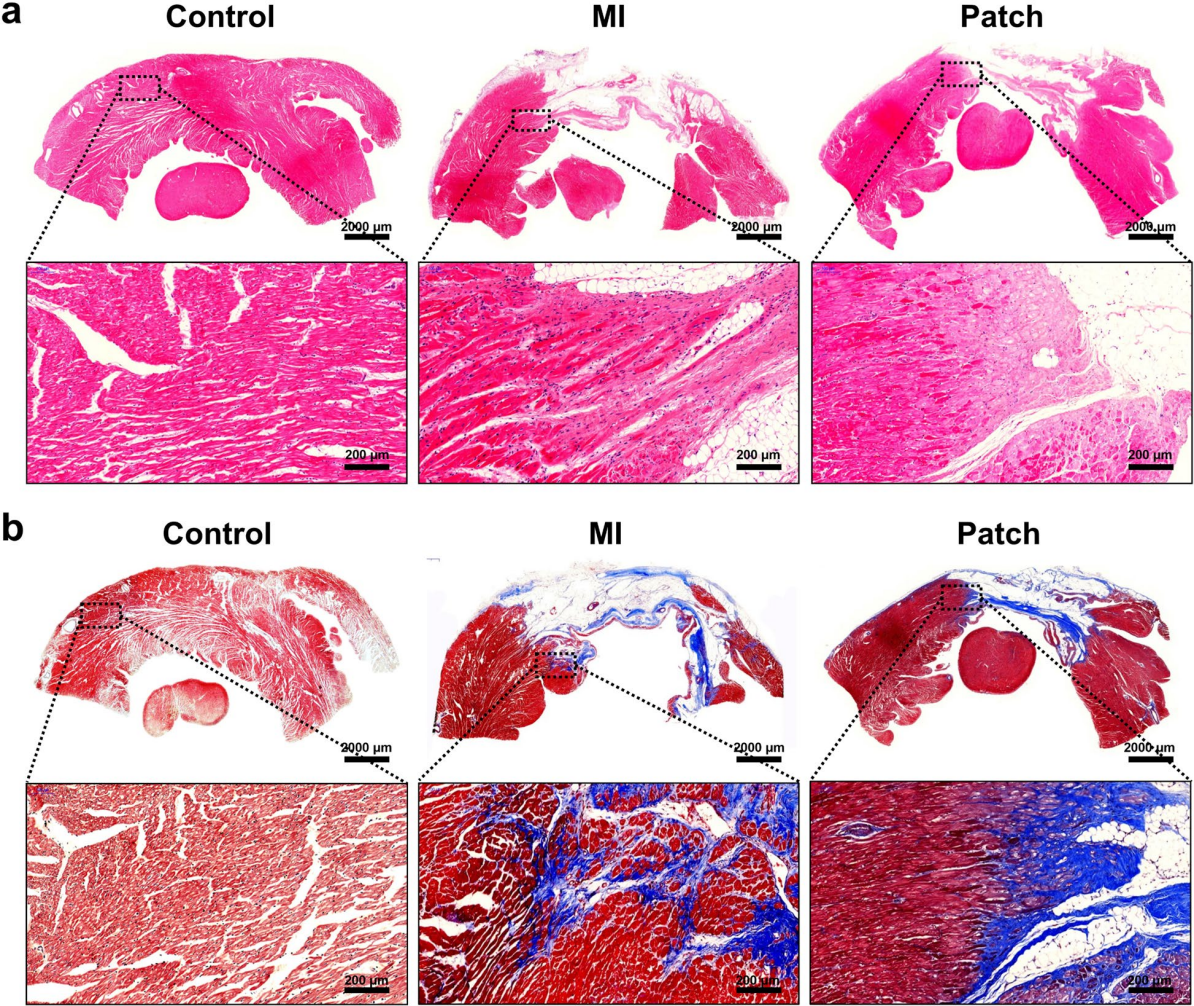
Representative histological images of heart cross sections: (**a**) H&E stain and (**b**) Masson’s trichrome stain (Scale bar, 2000 μm and 200 μm)

As the excellent proliferative effect *in vitro* with suitable physical features as fibrous patch at the concentration of 6% of collagen, this concentration of fibrous patch (6% Col/PLGA) was selected for further evaluation, and the *in vivo* test design included following three groups: “control group’, sham-operated group; rabbits without LAD ligation, “MI group”, those subjected to surgery but receiving no further treatments, and “patch treated group”, those treated with 6% Col/PLGA+ BMSCs. Figure 7a shows the representative images of heart cross sections subjected to H&E staining. In the control group, a striated cardiac muscle, myocardium, with the fairly normal nuclei were seen. In the groups of MI and patch, extensive necrotic tissues of a faint pinkish white were found. MI group showed that the myocardium was replaced by the irregular bands of fibrous tissues with the infiltration of macrophages along the edge of necrotic zone. This kind of tissue reaction was also evident in Masson's trichrome staining (Fig. 7b). Masson's Trichrome staining showed more intact collagen fibrous tissue (blue) compared with H&E staining that penetrates deeply into the myocardium tissues (red). This represents that acute myocardial infarction continues. Meanwhile, the group treated with patch showed somewhat different tissue responses. The myocardial fibers are not infiltrated by inflammatory cells nevertheless they are not normal either. The tissue between the fibers is not dense as in normal fibrous connective tissue (Fig. 7a). No further infiltration of collagen fibrous tissues into the myocardial tissues was not observed by MT staining (Fig. 7b). These represent a looser organizing type of connective tissue so called granulation tissue, otherwise known as an organizing stage of myocardial infarction. These staining also revealed decreased infarct size following BMSCs transplantation.

**Fig. 8:**
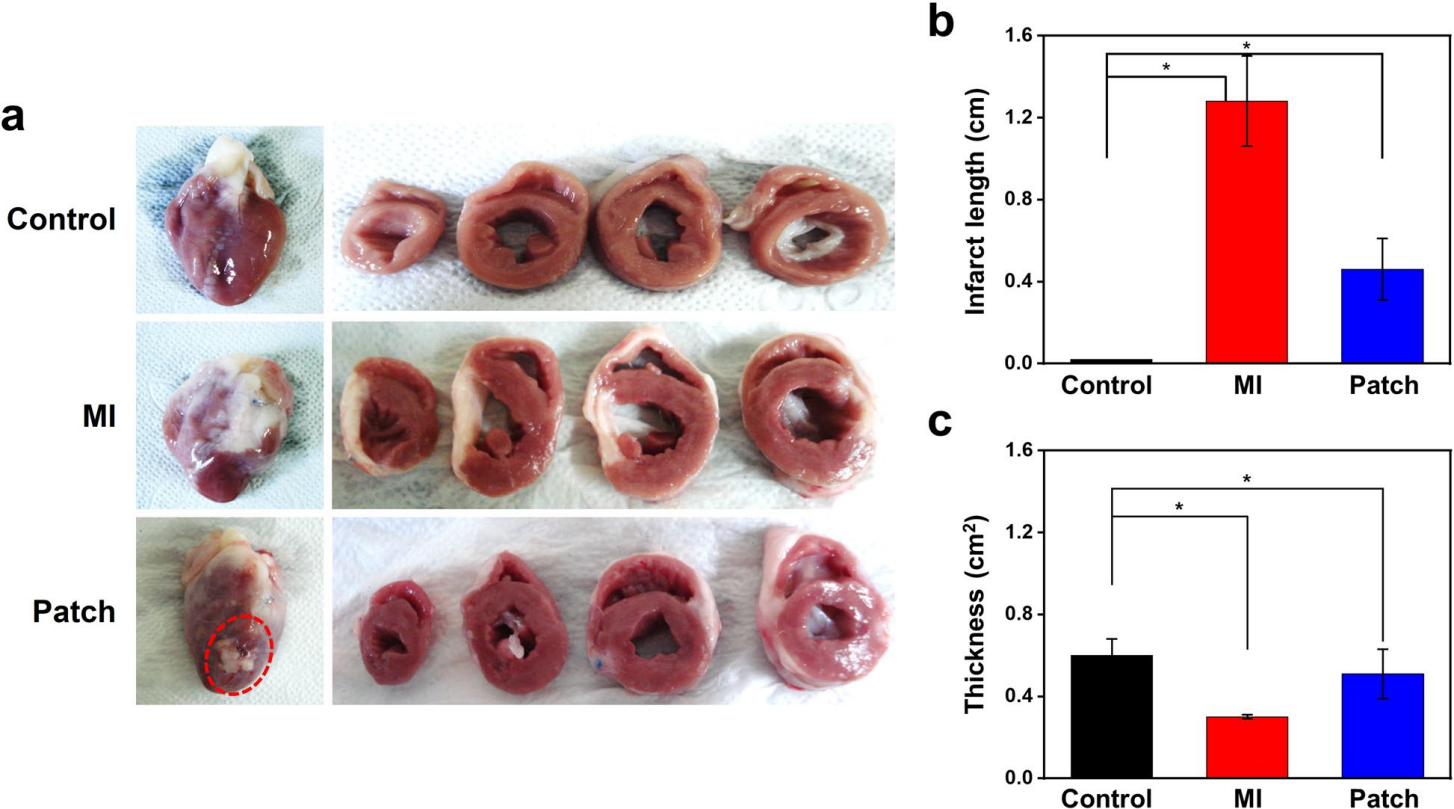
Representative images of sectional view of rabbit hearts (**a**) and measurement of infarct length (**b**) and LV wall thickness (**c**) (*p < 0.05). Red circle indicates remain patch in the heart tissue

Infarct length noticeably reduced about 3 times in the patch treated groups (0.46 ± 0.15 cm) compared to MI groups (1.28 ± 0.22 cm) (Fig. 8a). LV wall thickness increased approximately 2-fold in the patch treated group (0.51 ± 0.12 cm^2^) than in the MI group (0.24 ± 0.02 cm^2^) (Fig. 8b).

### The Apoptosis Analysis of the Myocardial Infarction

**Fig. 9:**
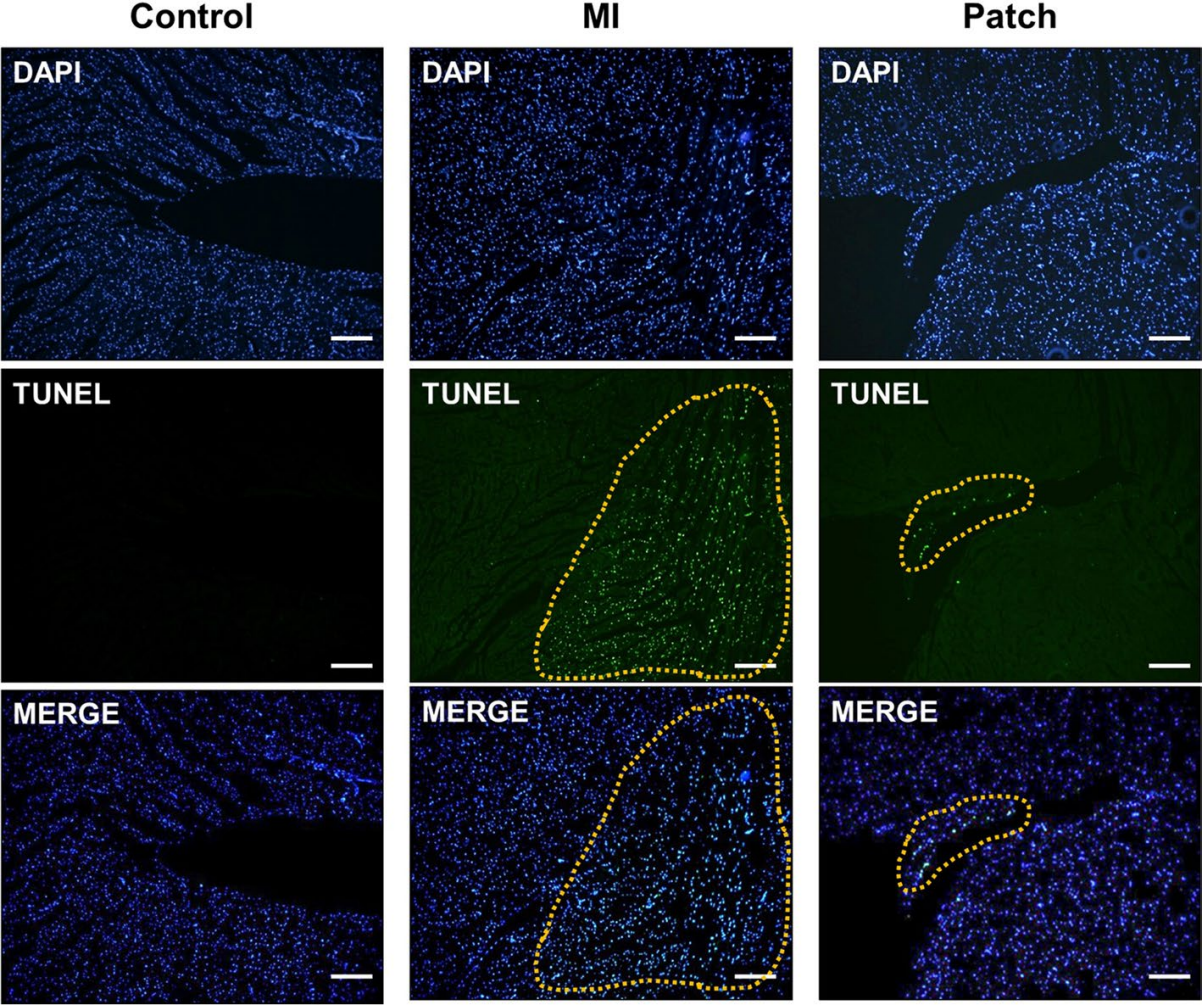
TUNEL assay for assessing cardiomyocyte apoptosis in the peri-infarct border zone: A huge number of apoptotic cells exist in the circled area (MI) markedly decreased in Patch treated group (Scale bar, 100 μm)

As the group treated with patch in H&E and Masson's Trichrome staining did not show continued progression of MI, we assessed cardiomyocyte apoptosis in the peri-infarct border zone using TUNEL assay (Fig. 9). The apoptotic cardiomyocyte were rarely found in the control group. At 4 weeks after left ventricular ligation, the MI group showed a large number TUNEL+ cells in the border zone, while the patch treated group showed significantly fewer numbers of TUNEL+ cells than MI group, suggesting that the cardiac patch has inhibitory effect on the progression of LV remodeling.

### *In vivo* tracing of fluorescent nanoparticle-labeled BMSCs in rabbit MI model

**Fig. 10:**
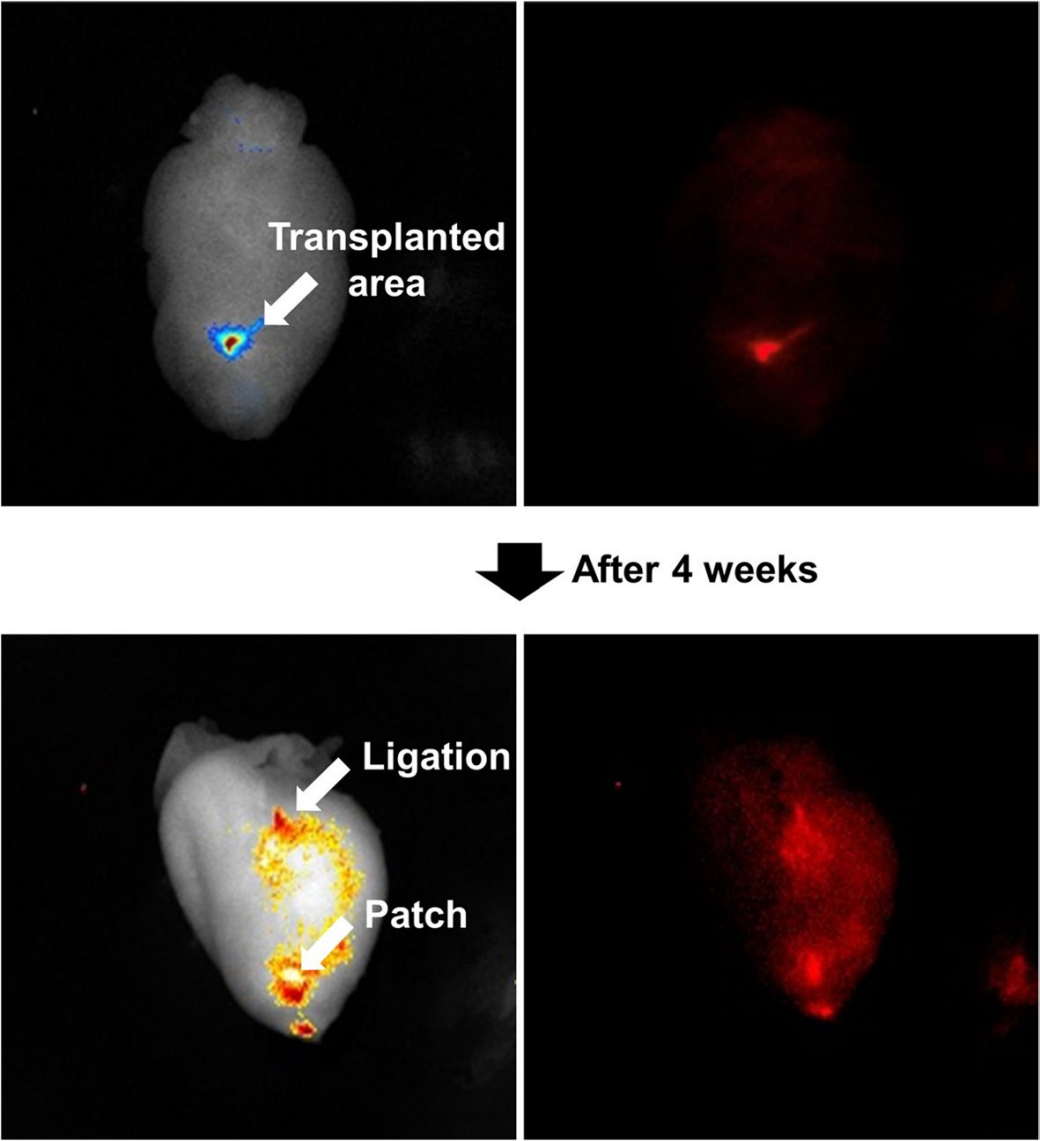
*In vivo* image of Patch treated rabbit heart. MSCs labeled with NEO-STEM (NIR797) migrated along LAD and eventually became infused to most of the myocardial tissue of the heart 4 weeks after transplantation

Labeling cells with NIR nanoparticles makes it possible to track transplanted BMSCs and monitor their migration. Using these advantages, the BMSCs retention capability of Col/PLGA patch was examined in rabbit MI model. At the beginning of transplantation, stem cells were found in the transplanted area, but after that, the stem cells migrated along LAD and eventually became infused to most of the myocardial tissue of the heart (Fig. 10).

### Immunohistochemistry

**Fig. 11:**
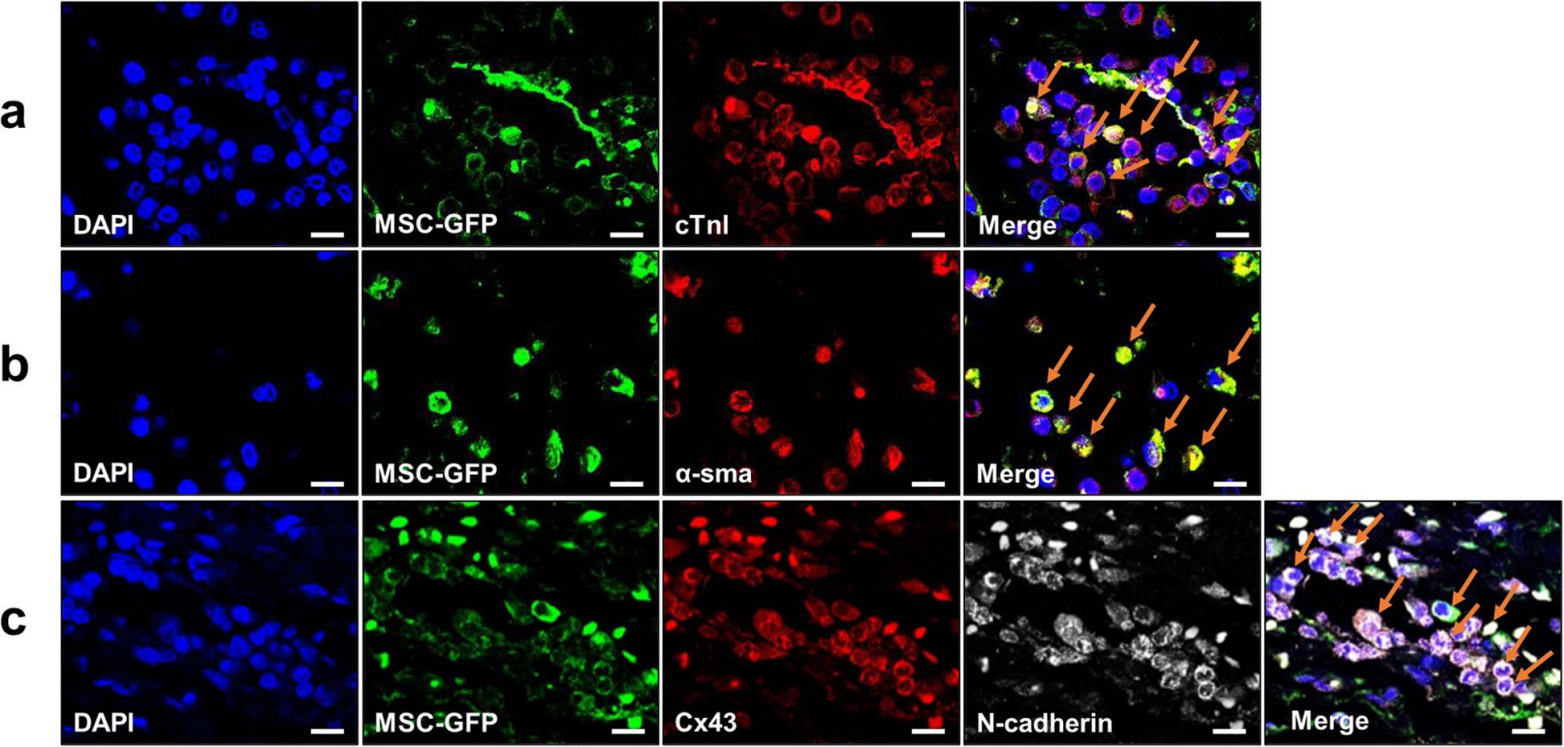
*In vivo* tracing tests of BMSCs via the treatment of border zone with GFP-BMSCs: (**a**) cTnI, (**b**) α-SMA and (**c**) N-cadherin and Cx43 (Scale bar, 10 μm)

In addition to *in vivo* tracking of nanoparticle-labeled BMSCs, we also traced the GFP-transfected BMSCs seeded in cardiac patch following *in vivo* transplantation [22]. And the expressions of myocardial cell-specific markers were confirmed using immunofluorescence staining in order to investigate whether the BMSCs migrated to the ischemic region and were associated with the therapeutic and recovery effects of myocardial regeneration (Fig. 11). Four weeks after transplantation, BMSCs in the patch migrated to the myocardium and then expressed cardio-regenerative markers (cTnI and α-SMA); those were double stained with GFP (arrows in the merged image) (Fig. 11 a, b). In addition, GFP-BMSCs showed a significant increase in N-cadherin and Cx43 expression at the cell-cell junction; appeared as appropriately integrated with the resident myocardium and with each other (Fig. 11c). These results strongly suggest that BMSCs migrated from the patch to the MI region, and were exhibiting strong cardiogenic potentials.

### Improved expressions of cardiac repair factors

**Fig. 12:**
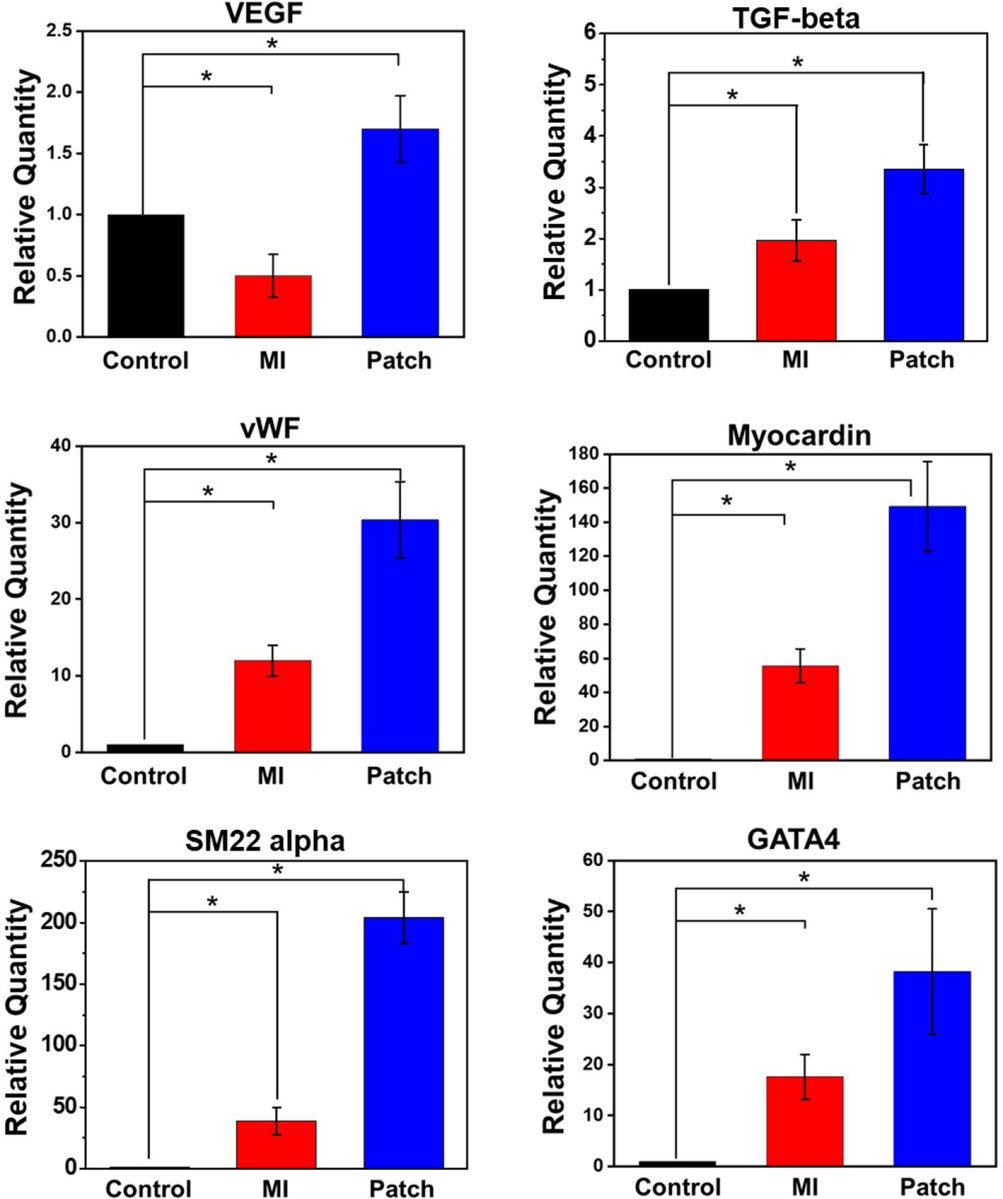
Determination of myocardial recovery factors through real-time PCR (*p < 0.05)

Figure 12 shows that the transplanted BMSCs improved cardiac ischemia by inducing angiogenesis due to the increased expression of VEGF and vWF. Moreover, the expression of myocardin, GATA-4 was highest in the patch treated group. The reason for the increase of these genes in the MI model appears to be due to the cardiac remodeling [23–26].

### Echocardiographic findings after Col/PLGA+ BMSCs transplantation in rabbit MI model

**Fig. 13:**
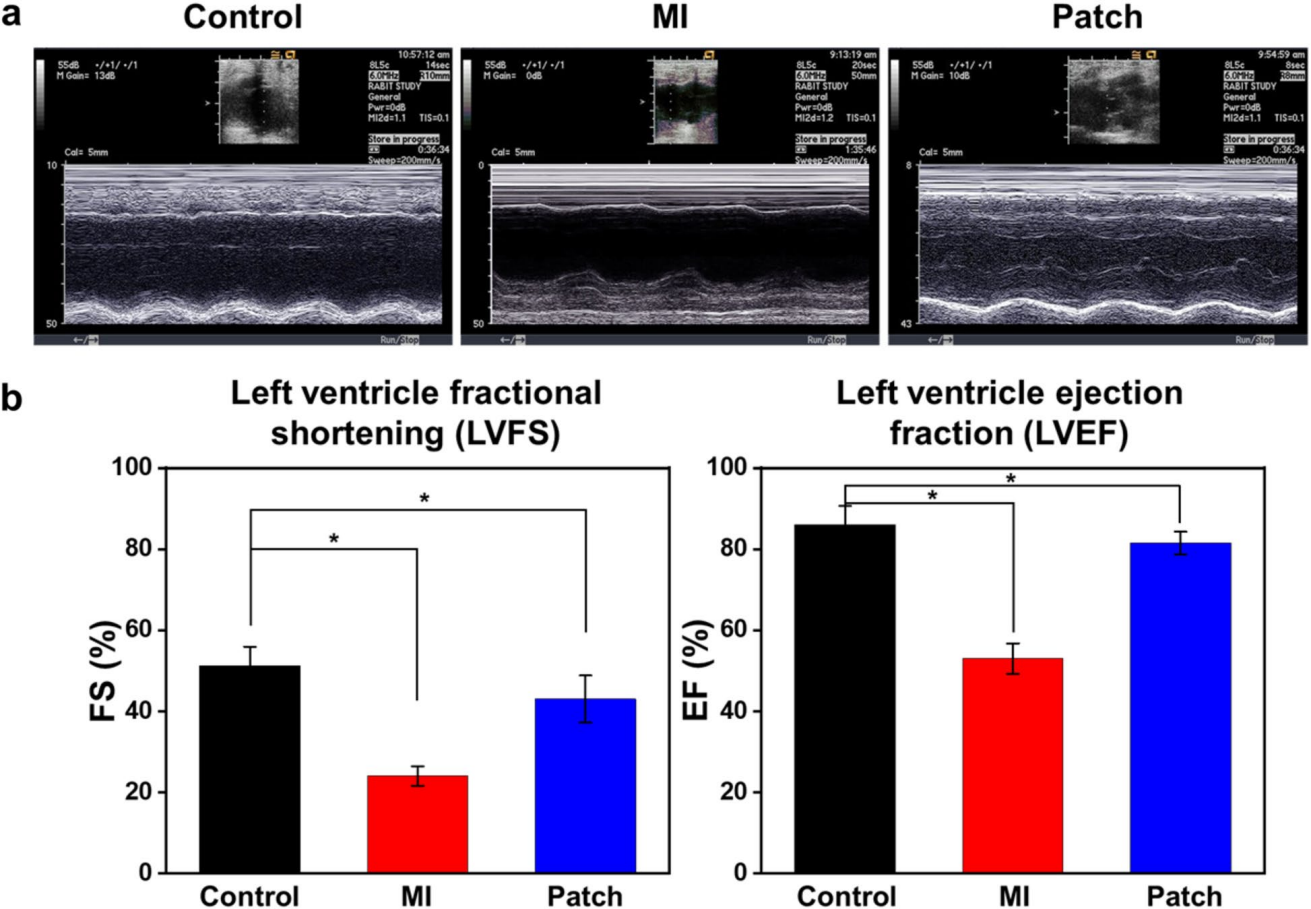
Echocardiographic measurement cardiac function of rabbits in different treatment groups (**a**) and their ejection fraction and fractional shortening analyzed based on the echocardiographic results (**b**) (*p < 0.05)

To examine the therapeutic efficacy of the cardiac patches, the echocardiographic assessments were carried out (Fig. 13). The left ventricular fractional shortening and left ventricular ejection fraction calculated through echocardiography confirmed that transplantation of BMSCs with cardiac patch markedly improved myocardial contractility (regenerative capacity) compared to MI. The ligation of LAD for MI model establishment significantly decreased the cardiac function. However, the patch group increased the LVFS and LVEF of the MI group by 191% and 158%, respectively. In particular, compared with the left ventricular fractional shortening (51.3%) and left ventricular ejection fraction (86.1%) of sham group, those of patch treated groups reached to 75% and 95% of normal values, respectively.

## Discussion

The use of cardiac patches is still controversial in that patches placed on the epicardium are often separated from the host myocardium by scar tissue [27]. However, cardiac patch has the significance in the field of the tissue engineered cardiac regeneration because it overcomes several shortcomings of intra-myocardial injection by providing a template for cells to form a cohesive sheet. So far, many biomaterials of natural and synthetic origin have been adapted for the manufacture of the engineered cardiac patches with various fabrication techniques [9, 28]. Among them, fibrous scaffolds fabricated using electrospinning technique have been increasingly explored for engineering functional cardiac tissues [29–31]. One of the problems with the use of electrospinning is that nanofibrous structures while highly bioactive, are nevertheless a barrier for cell migration into the scaffold [30–32]. As shown in Fig. S1, the pore sizes of the nano patches are distributed in the range less than 0.5 μm. Considering the size of BMSCs, it is almost impossible for the cells to migrate into the patch after focal adhesion. In this respect, we have prepared novel bi-modal electrospun scaffolds as a feasible strategy to address these challenges in cardiac tissue engineering. Nanoscale collagen fibers function as a cue for focal adhesion and spreading of cells, and microscale PLGA fibers incorporated micro/nano architecture that would increase the porosity of the fibrous scaffold and thereby improving the cell penetration, can be utilized for the development of large-scales cardiac constructs [30–32]. As shown in Fig. 3, the bi-modal scaffolds show remarkable increase in mechanical properties compared to collagen, which is also a prerequisite for suturing cardiac patch on epicardium (Scheme 1). In addition, bi-modal scaffolds showed the superiority in BMSCs proliferation compared to PLGA (Fig. 6).

To determine whether this highly proliferative bi-modal scaffold may function as a true cardiac patch, it is necessary to investigate that the seeded stem cells in scaffold are transplanted into ischemic sites with a high rate and long-term retention capacity. In this study, we employed long-term stem cells tracking systems for elucidating their fate, migration, and contribution to regenerating tissue. Figure 10 provides strong evidence for long-term engraftment of BMSCs transplanted through bimodal scaffolds. BMSCs labeled with NIR797 infused into the entire left ventricle wall 4 weeks after transplantation. BMSCs retention capability of cardiac patch was double confirmed by GFP transfection, and the immunohistochemical double staining offers indisputable evidence that long-term engrafted BMSCs possessed myocardial functions; numbers of GFP (green)-stained cells exist inside the heart wall, and many of them are double-stained with cardiac troponin-1 as well as α-smooth muscle actin, which are involved in the contraction of the heart wall or angiogenesis [33, 34] In addition, we found the co-localization region stained with connexin 43 and N-cadherin which are essential for formation of gap junction [35–37].

If the main effect of stem cell transplantation for cardiac regeneration is made through differentiation of stem cells into cardiac or smooth endothelial cells mentioned above, the second possible recovery can be made through paracrine effects that stem cells secret cytokines and growth factors which may promote angiogenesis, suppress cell death of resident cardiomyocytes, and modulate interstitial matrix composition. Therefore, real-time PCR was performed to confirm the paracrine secretion of BMSCs and their effects on the host tissue in RNA revel (Fig. 12). The strong expressions of BMSC paracrine factors (VEGF and TGF-beta) were found in the cardiac patch treated group compared with Control and MI group. As a result of increased expression of angiogenic paracrine factors (VEGF and TGF-beta), upregulated vWF and SM22 alpha indicate cardiovascular regeneration in the cardiac patch treated group [23, 24, 38–40]. Additionally, VEGF and TGF-beta are known as the key players in the regeneration of cardiac tissue. The marked expression levels of myocardin and GATA-4 can be explained by enhanced survival of cardiomyocytes and maintenance of cardiac function due to the upregulation of paracrine factors from the BMSCs [25, 26, 40]. Echocardiography demonstrated that these therapeutic results collectively affected the improvement of cardiac function. The two important parameters related to the left ventricular function, EF and FS values of rabbit hearts treated with cardiac patch were significantly higher than those of MI group, and they almost recovered to those of normal (Fig. 13). Consequently, nano and micro bimodal cardiac patches provided the basement for seeded stem cells to proliferate and the capability for long-term engraftment in the ischemic region, thereby allowing stem cells to function as myocardial and vascular endothelial cells or to secrete the recovery factors, which in turn led to improved heart function [41]. Such results thus indicated that cardiac patch could significantly attenuate cardiac remodeling and fully recover the cardiac function, as a consequence of their potent long term retention capability.

## Conclusions

In this study, a nano and micro bimodal composite fibrous scaffold composed of collagen and poly (D, L-lactic-co-glycolic acid) (Col/PLGA) was fabricated using an independent nozzle control multi-electrospinning apparatus, and its feasibility as the stem cell laden cardiac patch was investigated. Scanning electron microscopy (SEM) showed that nano/micro bimodal distributions of Col/PLGA without beaded fibers were obtained in the range of 4-6% of collagen concentration. The poor mechanical properties of collagen were improved by co-electrospinning with PLGA. Meanwhile, hydrophilic property of Col/PLGA was markedly increased by incorporation of collagen. *In vitro* experiments using BMSCs revealed that Col/PLGA showed improved cyto-compatibility and proliferation capacity compared to PLGA, and their extent increased with increase in collagen content. Through histological findings and echocardiography, *in vivo* studies demonstrated that BMSCs laden Col/PLGA dramatically promoted cardiac recovery. The results of tracing nanoparticle-labeled and GFP transfected BMSCs strongly support that Col/PLGA possesses the long-term stem cells retention capability, thereby allowing stem cells to directly differentiate into myocardial and vascular endothelial cells or to secrete the recovery factors, which in turn leads to improved heart function.

## Supplementary Information

**Additional file 1.**

## Data Availability

Data sharing in not applicable to this article.
