# Supplementary material for: Stem cell laden nano and micro collagen/PLGA bimodal fibrous patches for myocardial regeneration
Source: Biomater Res. 2022 Dec 13;26:79. doi: 10.1186/s40824-022-00319-w (PMC9749239; doi:10.1186/s40824-022-00319-w)
Supplement: Supplementary file 1 — Additional file 1. [file 40824_2022_319_MOESM1_ESM.docx]

**Supplementary Material**

**Stem cell laden nano and micro collagen/PLGA bimodal fibrous patches for myocardial regeneration**

Jung Hee Wee^1†^, Ki-Dong Yoo^2†^, Sung Bo Sim^3†^, Hyun Joo Kim^4†^, Han Joon Kim^5^, Kyu Nam Park^5^, Gee-Hee Kim^2^, Mi Hyoung Moon^6^, Su Jung You^4^, Mi Yeon Ha^4^, Dae Hyeok Yang^4^, Heung Jae Chun^4,7,8^*, Jae Hoon Ko^9^ and Chun Ho Kim^10^

*^1^Department of Emergency Medicine, Yeouido St. Mary’s Hospital, College of Medicine, The Catholic University of Korea, Seoul 07345, Republic of Korea*

*^2^Division of Cardiology, Department of Internal Medicine, St. Vincent’s Hospital, Suwon 16247, Republic of Korea*

*^3^Department of Thoracic and Cardiovascular Surgery, Bucheon St. Mary’s Hospital, College of Medicine, The Catholic University of Korea, Bucheon 14647, Seoul, Republic of Korea*

*^4^Institute of Cell and Tissue Engineering, College of Medicine, The Catholic University of Korea, Seoul 06591, Republic of Korea*

*^5^Department of Emergency Medicine, ^6^Department of Thoracic and Cardiovascular Surgery, Seoul St. Mary’s Hospital, College of Medicine, The Catholic University of Korea, Seoul 06591, Republic of Korea*

*^7^Department of Medical Life Sciences, ^8^Department of Biomedicine & Health Sciences, College of Medicine, The Catholic University of Korea, Seoul 06591, Republic of Korea*

*^9^Smart Textiles R&D group, Korea Institute of Industrial Technology (KITEC), Ansan 31056, Republic of Korea*

*^10^Laboratory of Tissue Engineering, Korea Institute of Radiological and Medical Sciences, 01812 Seoul, Republic of Korea*

^†^ These authors contributed equally to this work.

**^*^** Corresponding author, Heung Jae Chun

Professor and director

Institute of Cell & Tissue Engineering, College of Medicine, The Catholic University of Korea, Seoul, 06591, Republic of Korea

Tel: +82 2 2258 7033, Fax: +82 2 2258 7494, Email: chunhj@catholic.ac.kr


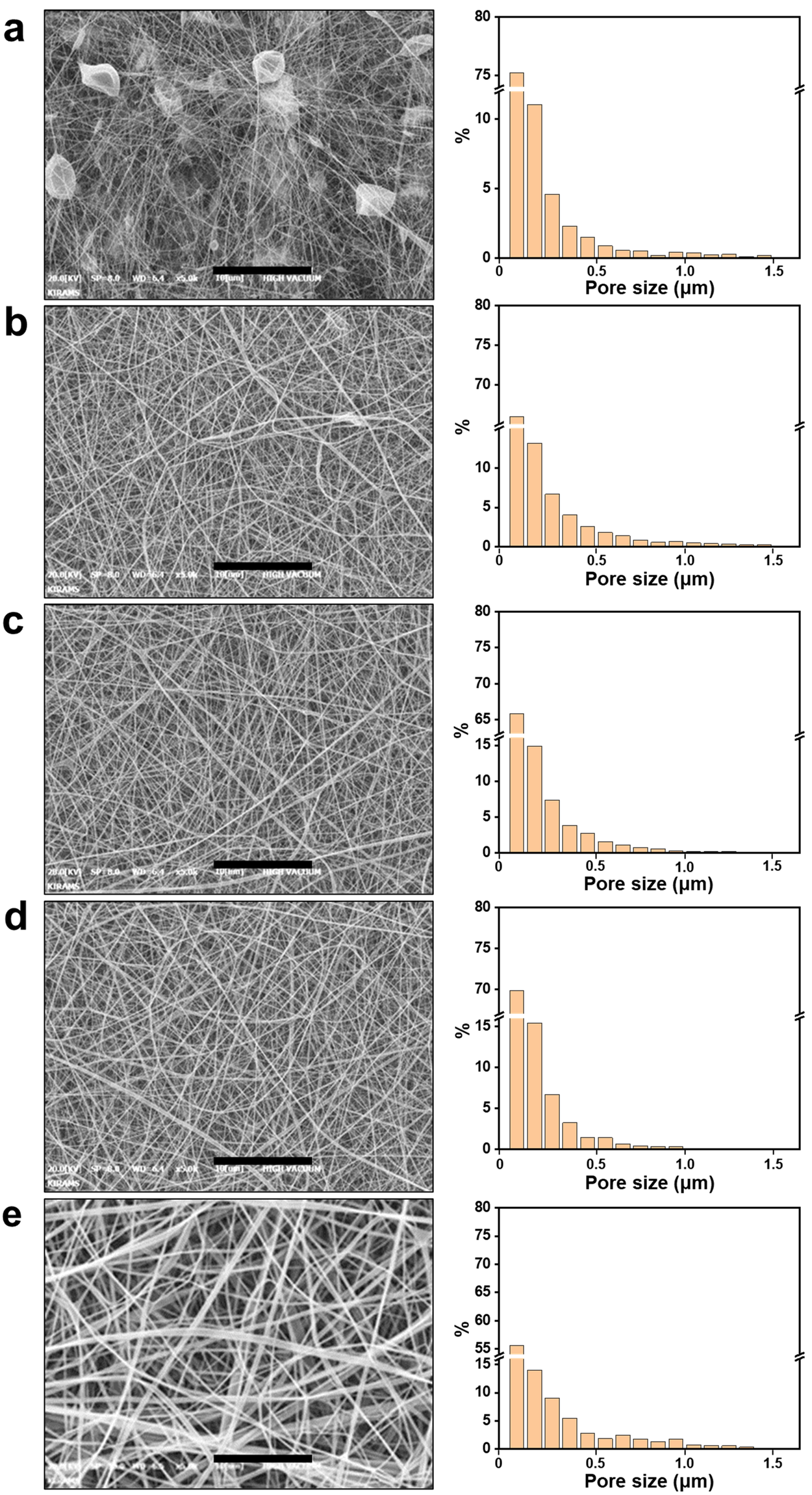


**Fig. S1** SEM images and pore size distribution of collagen fibrous samples (a) 3wt % collagen (b) 4wt % collagen (c) 5wt % collagen (d) 6wt % collagen (e) 7wt % collagen (Scale bar, 10 μm).
